# Intermittent Theta-Burst Transcranial Magnetic Stimulation Alters Electrical Properties of Fast-Spiking Neocortical Interneurons in an Age-Dependent Fashion

**DOI:** 10.3389/fncir.2016.00022

**Published:** 2016-03-30

**Authors:** Kathrin Hoppenrath, Wolfgang Härtig, Klaus Funke

**Affiliations:** ^1^Department of Neurophysiology, Medical Faculty, Ruhr-University BochumBochum, Germany; ^2^Rottendorf Pharma GmbHEnnigerloh, Germany; ^3^Pathophysiology of Neuroglia, Paul Flechsig Institute for Brain Research, University of LeipzigLeipzig, Germany

**Keywords:** repetitive transcranial magnetic stimulation, cortical fast spiking interneurons, parvalbumin, calcium-binding protein, development

## Abstract

Modulation of human cortical excitability by repetitive transcranial magnetic stimulation (rTMS) appears to be in part related to changed activity of inhibitory systems. Our own studies showed that intermittent theta-burst stimulation (iTBS) applied via rTMS to rat cortex primarily affects the parvalbumin-expressing (PV) fast-spiking interneurons (FSIs), evident *via* a strongly reduced PV expression. We further found the iTBS effect on PV to be age-dependent since no reduction in PV could be induced before the perineuronal nets (PNNs) of FSIs start to grow around postnatal day (PD) 30. To elucidate possible iTBS-induced changes in the electrical properties of FSIs and cortical network activity during cortical critical period, we performed *ex vivo*—*in vitro* whole-cell patch clamp recordings from pre-labeled FSIs in the current study. FSIs of verum iTBS-treated rats displayed a higher excitability than sham-treated controls at PD29–38, evident as higher rates of induced action potential firing at low current injections (100–200 pA) and a more depolarized resting membrane potential. This effect was absent in younger (PD26–28) and older animals (PD40–62). Slices of verum iTBS-treated rats further showed higher rates of spontaneous excitatory postsynaptic currents (sEPSCs). Based on these and previous findings we conclude that FSIs are particularly sensitive to TBS during early cortical development, when FSIs show an activity-driven step of maturation which is paralleled by intense growth of the PNNs and subsequent closure of the cortical critical period. Although to be proven further, rTMS may be a possible early intervention to compensate for hypo-activity related mal-development of cortical neuronal circuits.

## Introduction

Due to its modulatory action on human cortical excitability, repetitive transcranial magnetic stimulation (rTMS) has become a promising tool for the treatment of psychiatric and neurodegenerative disorders and cortical reorganization after stroke (Di Lazzaro et al., 2008a; Grefkes et al., 2010; Fitzgerald, 2011; for review, see Lefaucheur et al., 2014). However, little is known about the cellular mechanisms and network effects fundamental to the rTMS-induced changes in cortical excitability, a matter which appears as a prerequisite for optimizing the therapeutic potential of rTMS. The direction of change in cortical excitability primarily depends on stimulation frequency, with low frequencies (<5 Hz) being suppressive but higher frequencies being facilitative. Also the temporal structure of stimulation seems to be an effective parameter because raised cortical excitability is primarily found with intermittent stimulation, e.g., intermittent theta-burst stimulation (iTBS), while a single uninterrupted train (continuous TBS) tends to suppress cortical excitability (Huang et al., 2005). Besides possible induction of long-term depression (LTD)- and long-term potential (LTP)-like changes in cortical synaptic connectivity directly induced by low- and high-frequency stimulation protocols (Thickbroom, 2007), also a modulation of the activity of inhibitory cortical systems has been discussed on the basis of human data (Di Lazzaro et al., 2008b; Rogasch et al., 2014). By applying different rTMS protocols to rats, we could recently show that neuronal activity markers related to GABAergic neurons, like the 67 kD isoform of GABA synthesizing enzyme glutamic acid decarboxylase (GAD67) and the calcium-binding proteins parvalbumin (PV) and calbindin expressed in different classes of inhibitory interneurons (Kawaguchi and Kubota, 1998; Kawaguchi and Kondo, 2002; Markram et al., 2004), are differently affected by low- (1 Hz) and high-frequency (iTBS, cTBS) protocols and that particularly the iTBS protocol induces a strong and reliable reduction in the number of cells expressing PV within about 30 min (Trippe et al., 2009; Benali et al., 2011; Hoppenrath and Funke, 2013; Volz et al., 2013; Mix et al., 2014).

Neuropsychiatric disorders are discussed as being partly associated with a malfunction of inhibitory cortical systems, either as a result of acute interventions like abuse of drugs affecting glutamatergic transmission via NMDA receptors (Taylor and Tso, 2015) or as a result of prenatal disturbance of the development of inhibitory (cortical) systems (Selemon and Zecevic, 2015). In particular the number of interneurons expressing the calcium-binding protein PV is strongly decreased in patients with schizophrenic background (Beasley and Reynolds, 1997; Beasley et al., 2002; Lewis et al., 2005), accompanied by a diminished level of GAD67 (Knable et al., 2002; Hashimoto et al., 2003). Moreover, chondroitin sulfate proteoglycan-rich, polyanionic perineuronal nets (PNNs; Brückner et al., 1993) known to frequently surround PV^+^ interneurons (Härtig et al., 1992) are diminished in adolescents suffering from schizophrenia, indicating a discontinuous maturation of PNNs (Mauney et al., 2013). PNNs were recently discussed to be neuroprotective (Suttkus et al., 2016) and essential for long-term memory (Tsien, 2013). During neonatal cortical development PV^+^ fast-spiking interneurons (FSIs) play an important role in the activity-dependent fine-tuning of thalamocortical and some corticocortical synaptic connections—for example, PV^+^ interneurons are required for precise orientation tuning and binocular integration of sensory signals within the primary visual cortex (for review see Hensch, 2005). A manipulation of the sensory input like with dark-rearing or monocular deprivation in case of the visual system, or a pharmacological manipulation of the activity of the cortical inhibitory systems during this early cortical critical period considerably disturbs this process with long-lasting consequences in visual performance as known for amblyopia (Jefferis et al., 2015). Also prenatal disturbance of the development of inhibitory neurons may thus affect the plasticity processes of the cortical critical period and it would be of great advantage if this process could be positively affected by interventions like non-invasive brain stimulation.

In two recent studies, we already tested how iTBS, the protocol that strongly reduced the PV expression (40–50% reduction in the number of PV^+^ cells), affects the rat cortex when applied during the cortical critical period. Firstly, we found that the iTBS-induced decrease in PV expression could not be induced before postnatal day (PD) 30 but steeply increased between PD32 and PD37, paralleled by a strong burst of growth of the PNNs (Mix et al., 2015). This finding indicates that PV^+^ FSIs interneurons have to attain a distinct developmental state reached just during the critical period to respond to the stimulation. Secondly, we could show that iTBS applied during the critical period to dark-reared rats can prevent the loss of visual performance, obviously by enhancing the cortical brain-derived neurotrophic factor (BDNF) level in an activity-dependent fashion (Castillo-Padilla and Funke, 2016) that had been lowered by dark-rearing (Fagiolini et al., 1994). The current study was focused on the question whether PV^+^ interneurons respond to iTBS in an age-dependent fashion also by electrophysiological aspects. Therefore, we performed whole-cell *in vitro* patch-clamp recordings from pre-labeled FSIs of 26–62 days old rats to test them for changes in electrophysiological cell membrane properties and excitability, and further recorded spontaneous postsynaptic potentials to estimate changes in cortical network excitability.

## Materials and Methods

### Animals

Eighty-one male Sprague Dawley rats (Janvier, Genest-St-Isle, France) of different postnatal age (PD—postnatal day) were used in this study. Animals were kept in a 12 h day/night cycle (light on at 8:00 am) and had free access to food pellets and water. Rats increased in weight from about 50 g at PD26 to about 220 g at PD62. All animal experiments were performed with the permission of the government (AZ. 87-51.04.2010.A097) and the local animal welfare committee. All procedures complied with the guidelines of the animal welfare laws in Germany and the European Union.

### rTMS

**Figure 1:**
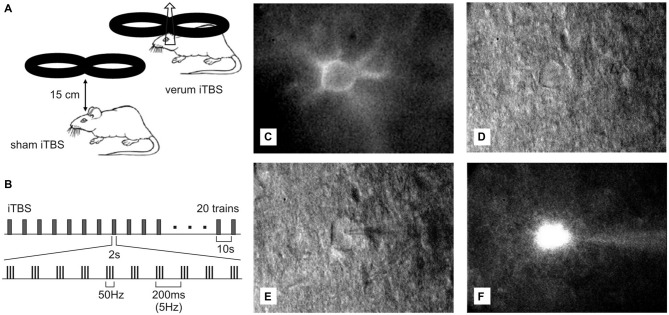
***Ex vivo*—*in vitro* whole-cell patch recordings of Cy3-*Wisteria floribunda* agglutinin (WFA)-labeled fast-spiking interneurons (FSIs) after transcranial magnetic stimulation (TMS). (A)** Drawing showing placement and orientation of the figure-of-eight coil used to apply the intermittent theta-burst (iTBS) protocol (see **B**) to the rat brain. At the orientation shown, a mediolaterally oriented electric field is induced within the brain, optimally suited to depolarize axons of the corpus callosum. **(C)** Red fluorescent Cy3-WFA-labeled perineuronal net of a cortical FSIs revealed by fluorescence microscopy. **(D)** The same neuron shown in infrared phase contrast illumination. **(E)** Same as in **(D)** but with the patch pipette attached to the cell. **(F)** Cell filled with Alexa488.

As in previous studies (Mix et al., 2010; Hoppenrath and Funke, 2013), rTMS was applied to conscious rats which had been familiarized to the stimulation procedure during the week prior to sham or verum stimulation. Rats were trained to tolerate the manual restraint and the noise and skin sensations (tingling) related to the rTMS procedure to avoid effects due to acute stress. The animal’s body was kept with both hands and with the index fingers flanking the head to achieve a stable head-to-coil position. Food rewards served as positive reinforcements. According to previous experiments (Mix et al., 2010, 2015; Hoppenrath and Funke, 2013), rTMS was applied below motor threshold (threshold to induce motor responses) using 23% of maximal machine power. Stimuli were generated with a MagStim rapid^2^ magnetic stimulator (The MagStim Company, Whitland, Dyfed, UK) and applied via a conventional 70 mm figure-of-eight coil positioned tangential to the dorsal surface of the rat’s head at a distance of about 8 mm in case of verum stimulation but 150 mm in case of sham stimulation. Direct coil-to-skin contact was avoided and the absolute distance between head and coil was slightly adjusted to just prevent muscle twitches. As previously described, we used a coil orientation capable of inducing a mediolaterally oriented electric field within the brain, suitable to activate primarily axons of the corpus callosum but less other structures of the brain directly (see Benali et al., 2011; Mix et al., 2015). Thereby, we likely achieved synaptic activity in cortical layers 2/3 via the axon collaterals of callosal axons, suitable to mimic activation patterns in human cortex. Each animal received a total number of 1800 pulses iTBS, applied as three blocks of the conventional iTBS protocol (Huang et al., 2005) separated by 15 min (Nettekoven et al., 2014). One iTBS block consisted of 20 trains with each train including 10 bursts of three pulses at 50 Hz which were repeated at 5 Hz. Each trains lasted 2 s and was repeated at 10 s intervals, summing up to a total block length of 192 s (see also Figures 1A,B).

### Slice Preparation

Brain slices including the somatosensory cortex were prepared as frontal sections (SC, 1.8 mM posterior to bregma, 290 μm thick). One hour after the last iTBS block rats were deeply anesthetized with carbon dioxide and killed by decapitation. The brains were quickly removed and stored in ice-cold artificial cerebrospinal fluid (ACSF). Six coronal slices were prepared from each brain using a vibratome (VT 1000S, Leica, Wetzlar, Germany). Slices obtained from animals up to PD38 were incubated in ACSF containing: NaCl 125 mM, KCl 3 mM, NaHCO_3_ 26 mM, CaCl_2_ 2.5 mM, MgSO_4_ 1.3 mM, NaH_2_PO_4_ 1.25 mM, D-glucose 13 mM, pH 7.4, continuously oxygenated with carbogen (95% O_2_, 5% CO_2_). Sections from animals older than 40 days were incubated in a cutting solution containing a somewhat different composition of substances to prevent excitotoxicity: NaCl 87 mM, NaHCO_3_ 26 mM, D-glucose 10 mM, sucrose 75 mM, KCl 2.5 mM, NaH_2_PO_4_ 1.25 mM, CaCl_2_ 0.5 mM, MgSO_4_ 3.5 mM, pH 7.4. All slices were kept in oxygenated ACSF for at least 30 min at 30°C before recording. To label PNNs surrounding PV expressing FSI in the living brain slice, 20 μl of Cy3-tagged *Wisteria floribunda agglutinin* (WFA; 1.8–2 mg/ml; prepared according to Brückner et al., 1996) was added to 2 ml ACSF, and with the brain slices incubated for up to 1 h at room temperature under continuous oxygenation. In pilot experiments, with variations in the concentration of Cy3-WFA, incubation time and temperature, and in comparison to bolus injections into the slice, we found these settings to be most effective for sufficiently labeling the PNNs of FSIs within 1 h and devoid of high levels of background staining. Next, single slices were transferred to the recording chamber, mounted on an upright epifluorescence microscope (OLYMPUS-BX51-WI, Olympus Deutschland GmbH, Hamburg, Germany) equipped with a 4× and a 40× water immersion objective. The recording chamber was steadily perfused at a rate of 1.25 ml/min with oxygenated ACSF of 33°C.

### Electrophysiological Recordings and Data Analysis

Patch electrodes were prepared from 1.5 mM borosilicate glass capillaries (GB 150F-8P, Science Products GmbH, Germany) using a horizontal puller (P-1000, Sutter Instruments Co., Novata, CA, USA) with settings resulting in 6–9 MΩ total impedance. The potassium-based internal solution contained Alexa488 hydrazide (100 μM, Molecular Probes, Eugene, TX, USA) dissolved in the following mixture (in mM): K-gluconate 97.5, KCl 32.5, HEPES 10, MgCl_2_ 1, Na_2_ATP 4, EGTA (ethylene glycol tetraacetic acid) 5, at pH 7.3 (adjusted with KOH). In case of measuring postsynaptic currents, 5 mM QX-314 chloride (N-Ethyllidocaine chloride) was added to the pipette solution to prevent action potentials by blocking the voltage-activated sodium channels. Cy3-WFA-labeled interneurons were visualized and detected using epifluorescence illumination in combination with infrared-differential interference contrast (IR-DIC) microscopy (see Figures 1C–F) and a filter setting suitable for detection of Cy3 and Alexa488 fluorescence. Single cell responses obtained with whole-cell patch clamp recordings were amplified using a PC-501A amplifier (Warner Instruments, Hamden, CT, USA), recorded and digitized at a sampling frequency of 2.73 kHz using WinWCP Software (version 4.6.7, Strathclyde University, Scotland) and a Digidata 1440A interface (Axon CNS, Molecular Devices, Sunnyvale, CA, USA) before being finally stored on a hard disk for off-line analysis. Since rTMS effects fade with time, we applied whole-cell patch recordings instead of perforated patch recordings because the latter require a further equilibration phase of 30–40 min until the number of gramicidine pores stabilizes. To minimize dilution of cytoplasmatic PV with pipette solution, recordings were kept as short as possible (~10 min) by measuring current injection induced action potential firing and spontaneous synaptic currents in different cells. Thereafter, patch-clamps were directly terminated for analysis another cell in the same or a different slice. Unfortunately, this procedure of short whole-cell patch recordings yielded most cells insufficiently stained with Alexa488 for histological verification.

After the whole-cell configuration had been achieved in voltage clamp mode, 10 voltage pulses of 100 ms duration (−100 mV to −10 mV at steps of 10 mV) originating from a holding potential of −60 mV were applied to obtain input resistance, time constant and capacitance of the cell membrane from the current-voltage relationships. Subsequently, while in current clamp mode, somatic currents of −50 pA to +115 pA and 100 ms duration were injected to reveal membrane resting potential (MRP) and input resistance. A depolarizing current ramp of 1 mV/ms induced by 80 pA current injection was used to evoke action potentials and to determine the threshold of action potentials. In order to analyze evoked spike firing, five different depolarizing current steps (100–500 pA, step size 100 pA) were injected for 1 s into one sample of cells. Data were analyzed as spiking frequency vs. injected current plots. Measurements of spontaneous postsynaptic currents (sPSCs) were done in a separate sample of cells at −80 mV holding potential during five recording episodes of 2 s each. Measurements were performed twice, without and with 100 μM picrotoxin (PTX, Tocris) added to the bath solution to block GABA_A_ receptor mediated spontaneous inhibitory postsynaptic currents (sIPSCs), while leaving the spontaneous excitatory postsynaptic currents (sEPSCs) unaffected. For detection and analysis of sPSCs, we used WinEDR Software (Strathclyde University, Glasgow, UK) and Mini Analysis Software (Synaptosoft Inc. Decatur, GA, USA). Threshold for sPSC detection was set to 2-times the standard deviation of noise amplitude and further parameters like initial rise in sPSCs and duration were adjusted for optimal sPSC detection under visual inspection using Mini Analysis software. sPSCs resulting from temporal summation of sPSCs were also manually excluded from the statistical analysis. In addition, WinWCP (Strathclyde University) and Clampfit 10.2 (Axon Instruments, Molecular Devices, Sunnyvale, CA, USA) Software were utilized for recordings and data analyses. Generally, one cell was studied per slice if measurements were successful and slices were refused if no stable whole cell patch-clamp recordings could be achieved within 1 h after Cy3-WFA pre-labeling.

### Histochemical Analysis

Following electrophysiology brain slices were transferred to 4% paraformaldehyde for 48 h. After cryoprotection in 30% sucrose for 3 days, 30 μm thick coronal cryosections (parallel to brain slice surfaces) were prepared and directly mounted on slides for visual inspection in a fluorescence microscope (Leitz Wetzlar Dialux 20 microscope; Leica, Solms, Germany). Sections containing Alexa488 stained cells were then used for immunohistochemical staining of PV while the PNNs were labeled with Cy3-conjugated Cy3-WFA. After a first washing procedure using phosphate buffered saline (PBS), slices were blocked for 90 min in 10% normal goat serum in PBS containing 0.2% Triton X-100. The free-floating slices were incubated overnight at room temperature with rabbit-anti-PV (1:500, Swant, Marly, Switzerland) diluted in PBS containing 1% normal goat serum and 0.2% Triton X-100. Additionally, this solution contained Cy-3-WFA (*ca*. 20 μg/ml, prepared according to Brückner et al., 1996) to label PNNs. Thereafter, Alexa647-tagged donkey-anti-rabbit IgG, 1:500 in PBS containing 1% normal goat serum and 0.2% Triton X-100 (Jackson ImmunoResearch, West Grove, PA, USA) was applied for 90 min at room temperature. After a final washing procedure, slices were mounted on gelatinized object slides, dried overnight at 4°C and coverslipped with Depex (Serva, Heidelberg, Germany). All steps were performed in the dark to avoid fading of the fluorescent dyes. Images were taken at a final magnification of 20× and 63× using a fluorescence microscope (Apotome, Zeiss, Jena, Germany).

### Statistics

All data sets were first tested for normal distribution using the Kolmogorov-Smirnov test. Those showing normal distribution (induced spike-firing rates; MRP, input resistance, capacitance, time-constant and spike threshold, sEPSC frequency) are expressed as mean ± SEM and were analyzed using analysis of variance (ANOVA) and *post hoc* Bonferroni test (or 2-sided *t*-test if only 2 groups were compared). sPSC amplitudes showing non-normal distribution were analyzed with Kruskal-Wallis test if more than two groups were compared, otherwise Wilcoxon rank-sum test had been applied. All statistical tests were performed using IBM SPSS statistical analysis package version 22.0 and considering a *p*-value less than 0.05 as a criterion to reject the null-hypothesis.

## Results

**Figure 2:**
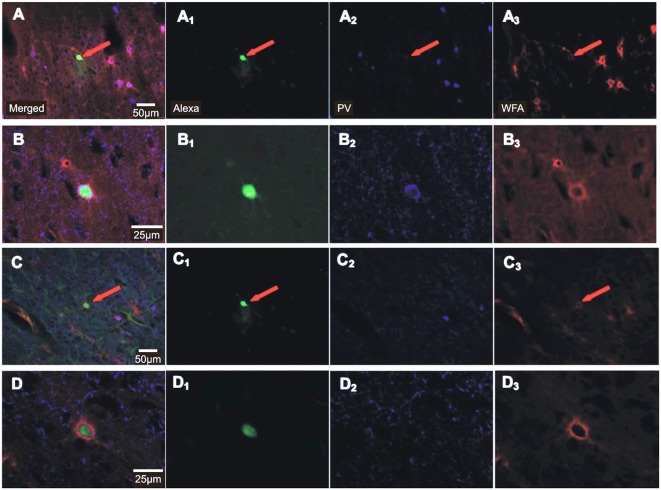
**Histochemical staining of recorded FSIs for Alexa488, parvalbumin (PV) and Cy3-WFA.** Photomicrographs of 30 μm-thick sections obtained from the 290 μm thick brain slices after recordings show Alexa488 stain (green), parvalbumin (PV, Alexa647, blue) and WFA (perineuronal nets, Cy3, red) of two different FSIs **(A–D)** at different magnification (20× in **(A)** and **(C)**, 63× in **(B)** and **(D)**). Merged images to the left **(A–D)**, individual stains to the right **(A_1_–D_3_)**. The red arrows indicate labeling in the images of lower magnification. Notice, that the cell indicated by an arrow in **(A)** was PV-immunoreactive whereas the marked cell in **(C)** was PV-immunonegative.

In total, 184 Cy3-WFA labeled cells were recorded in the whole-cell patch mode. Of these, 114 neurons were finally included in the analysis of induced spike-firing and a different set of 52 neurons was analyzed for spontaneous post-synaptic potentials (sPSPs), cells for which all test protocols could be successfully finished without signs of changes in the electrical stability (stable access resistance throughout recordings) and physiology of the cells. The majority of these cells showed the typical non-adapting firing characteristics of FSI, with firing frequencies increasing with current injection up to 300 spikes/s (see Supplementary Figure 1). The mean spike adaptation coefficient, calculated as the ratio of spike frequency close to the end of the current injection (750–950 ms) to spike frequency at onset of current injection (0–200 ms), was within the range reported earlier for FSI (e.g., Imbrosci et al., 2014). We deliberately did not narrow the range of adaptation coefficients and did not further exclude cells to avoid a selection bias that could hamper the rTMS effect. During whole-cell patch recordings cells were passively filled with Alexa488 for subsequent histochemical characterization using Cy3-WFA and anti-PV antibodies. However, due to weak staining in most cells only a few Alexa488-filled neurons could be visualized. All showed Cy3-WFA staining of surrounding PNNs and part of them displayed PV-labeling (for two examples, see Figure 2). A quantitative analysis regarding PV content became unrealistic due to the small number of stained cells and the possible dilution of the cytoplasm of FSI during patch-recordings.

### iTBS Effects on Induced Spike Firing

We have previously shown (Mix et al., 2015) that the effect of iTBS, the lowering of PV expression in neurons ensheathed by Cy3-WFA-staining, is age-dependent, first evident at PD30–32 when the PNNs of FSI start to mature. Therefore, we investigated the iTBS effects on the electrical properties of FSI in animals spanning this critical age period (PD26–38) and additionally in somewhat older animals (PD40–42 and PD60–62). Since not enough cells could be successfully studied per PD in both groups (sham, verum iTBS), a grouping including data of three consecutive PD appeared appropriate to achieve reasonably balanced cell numbers per group (sham/verum): PD26–28, 6/9; PD29–31, 8/13; PD32–35, 6/8; PD36–38, 6/10; PD40–42, 11/18; PD60–62, 6/13.

**Figure 3:**
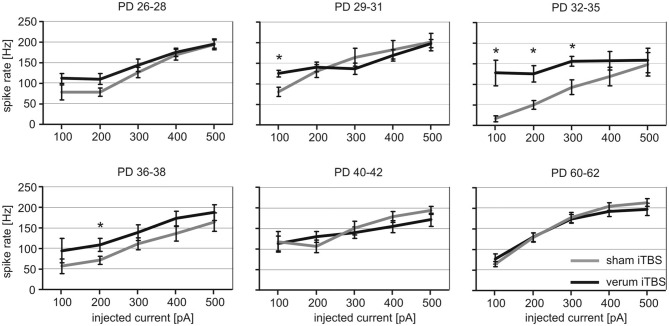
**Quantitative analysis of verum vs. sham intermittent theta-burst stimulation (iTBS) induced spike-firing for FSI tested at different age.** The diagrams show mean rate of spike firing (spikes/s ± SEM) of FSIs induced by current injections between 100 and 500 pA for animals of different age (postnatal days, PD) which had received either sham (gray lines) or verum (black lines) iTBS. *p < 0.05 (*t*-test, 2-sided). Number of analyzed cells per age group (in ascending order of age): sham 6, 8, 6, 6, 11, 6; verum 9, 13, 8, 10, 18, 13.

FSI of sham and verum iTBS treated rats showed the typical increase in spike firing rate with increased current injections, approaching 150–250 Hz with 500 pA. However, cells of PD26–38 obtained from verum iTBS treated rats showed a higher induced spike firing rate than sham-treated animals with low injection currents (100–300 pA) while no difference was found for PD40–42 and PD60–62. The effect was strongest for FSI of PD32–35 (Figure 3). ANOVA calculation with the three factors AGE (6 groups), TMS (sham, verum) and CURRENT (100–500 pA) revealed a significant effect of all factors (AGE: *F*_(5)_ = 4.48, *p* = 0.001; TMS: *F*_(1)_ = 12.37, *p* < 0.001; CURRENT: *F*_(4)_ = 52.798; *p* < 0.001). *Post hoc* pairwise comparisons (Bonferroni) showed significant differences in induced spike-firing between all current injections except for the difference between 400 and 500 pA, indicating a saturation effect with strong stimulation. Significant interactions between factors AGE and TMS (*F*_(1,5)_ = 4.095; *p* = 0.001) as well as between AGE and CURRENT (*F*_(5,4)_ = 1.736; *p* = 0.025) indicate an age- and stimulation-dependent TMS effect. This was verified by a pair-wise comparison of sham and verum treated groups, revealing significant differences for the following age groups and current injections: PD29–31 with 100 pA (sham 81 ± 10 Hz vs. verum 125 ± 8 Hz, *p* = 0.01), PD32–35 with 100, 200 and 300 pA (100 pA: sham 15 ± 7 Hz vs. verum 127 ± 31 Hz, *p* = 0.02; 200 pA: sham 49 ± 10 Hz vs. verum 124 ± 19 Hz, *p* = 0.01; 300 pA: sham 92 ± 17 Hz vs. verum 155 ± 12 Hz, *p* = 0.01) and PD36–38 with 200 pA current injection (sham 71 ± 9 Hz vs. verum 108 ± 16 Hz, *p* = 0.03). The regularity of induced spike sequences—quantified by the variance of inter-spike intervals—did not change as verified by pair-wise comparison of sham and verum groups as done for mean firing frequency (*p* > 0.3 for all pairs).

### iTBS Effects on Passive Cell Membrane Properties and Action Potential Threshold

**Figure 4:**
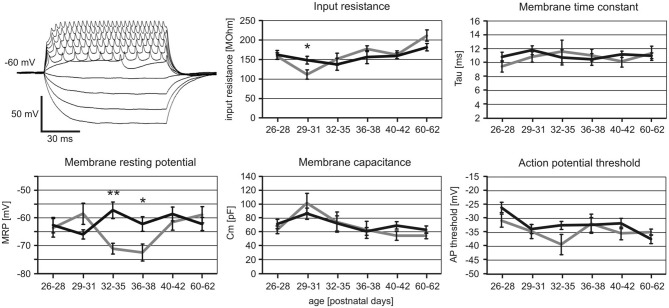
**Changes in cell membrane characteristics of sham and verum iTBS-treated rats of different age.** Example voltage traces obtained with −50 to +115 pA current injections to determine membrane resting potential (MRP) and input resistance are shown to the upper left. Other membrane parameters were determined in voltage clamp mode, testing −100 mV to −10 mV at steps of 10 mV. Diagrams show mean values (± SEM) of MRP, input resistance, time constant, capacitance and action potential threshold plotted vs. age for sham (gray) and verum (black) iTBS-treated animals. *p < 0.05, **p < 0.01 (*t*-test, 2-sided). Number of analyzed cells per age group (in ascending order of age): sham 8, 8, 6, 7, 12, 6; verum 11, 15, 9, 10, 25, 15.

We further analyzed whether iTBS leads to changes of the passive membrane properties of FSIs or the threshold for eliciting action potentials in them. As visualized in Figure 4, only the MRP showed clear differences between verum and sham-treated animals in the age groups PD32 to PD38. ANOVA resulted in a significant interaction of factors AGE and TMS (*F*_(1,5)_ = 3.017, *p* = 0.013) and *post hoc* comparison of factor TMS revealed significant differences in MRP for PD32–35 (sham −71 ± 2 mV vs. verum −57 ± 3 mV, *p* = 0.009) and PD36–38 (sham −72 ± 3 mV vs. verum −62 ± 2 mV, *p* = 0.03). No other comparisons revealed significant group differences. The more depolarized MRP of cells of the verum group appears to be primarily related to a more hyperpolarized level in the sham-treated rats at this age compared to younger and older animals. Cross-reference to induced spike-firing (Figure 3) reveals that also firing rate induced by weak currents is lower around this age (PD32–28) compared to younger and older animals of the sham group (100 pA, *p* < 0.05). Other cell membrane properties like input resistance, time constant, capacitance and action potential threshold appeared not to be affected by iTBS. Only the input resistance showed a marginally significant difference for the PD29–31 groups (sham 111 ± 12 MΩ vs. verum 148 ± 10 MΩ, *p* = 0.04).

### iTBS Effects on sPSCs

**Figure 5:**
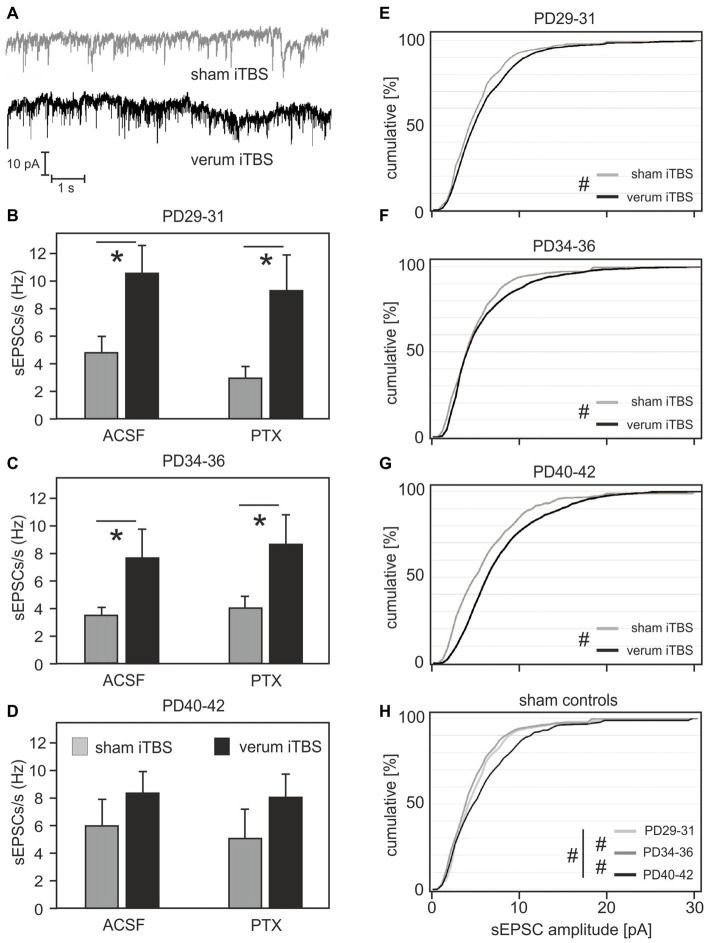
**Frequency and amplitudes of spontaneous postsynaptic currents (sPSCs) in FSIs of sham or verum iTBS-treated rats of different age. (A)** Example traces showing sPSC recordings in FSIs of sham (gray) and verum (black) iTBS-treated rats at PD35. **(B–D)** Bar diagrams show mean frequency of sPSCs (± SEM) for sham (gray) or verum (black) iTBS-treated rats of three different age ranges. Total sPSCs including excitatory (sEPSCs) and inhibitory (sIPSCs) currents were recorded with the GABA_A_ receptor blocker picrotoxin (PTX) not added to the bath solution (ACSF, left columns). sEPSCs were selected by blocking sIPSCs with PTX (right columns) which resulted in little reduction in sPSC rate. **(E–G)** Cumulative plots of sEPSC amplitudes for animals of different age having received either sham (gray) or verum (black) iTBS. Verum iTBS causes a shift towards larger sEPSCs primarily in the older animals (PD40–42). **(H)** Cumulative plot of sEPSC amplitudes for the sham-control animals of different age. sEPSC amplitudes increased between PD34–36 and PD40–42. *p < 0.05 (*t*-test, 2-sided), ^#^*p* < 0.001 (Wilcoxon rank-sum test).

The analysis of spontaneous synaptic currents was performed on a different set of animals and Cy3-WFA-labeled cells to prevent changes in the physiological conditions of the small FSI due to long recording times. Only rats aged 29–42 days were included, divided into three groups to address the range of age with the strongest iTBS effects on induced spike-firing and MRP at the transition of the former groups PD32–35 and PD36–38 (PD34–36; sham: *N* = 10, verum: *N* = 8), as well as the flanking younger (PD29–31; sham: *N* = 11 cells, verum: *N* = 8) and older former groups (PD40–42; sham: *N* = 8, verum: *N* = 7). After cells had been verified as fast-spiking, using a 300 pA injection current (as described above), sPSCs were recorded at a holding potential of −80 mV, first without, then with the GABA_A_ receptor blocker PTX added to the bath solution (Figure 5A). Since the pipette was filled with a solution containing 34.5 mM chloride, the calculated reversal potential for chloride currents was about −35 mV, thus guaranteeing sufficient potential difference to drive sIPSCs at a holding potential of −80 mV. Therefore, the first set of measurements without PTX included sEPSCs and sIPSCs of same current polarity, while the second measurements with PTX added to the bath solution should be free of sIPSCs. A group comparison using ANOVA with factors AGE, TMS and PTX revealed a significant effect only for factor TMS (*F*_(1)_ = 21.854; *p* < 0.001). *Post hoc* comparison of the TMS groups yielded significantly enhanced sPSCs rate (sEPSCs + sIPSCs) with iTBS only in animals aged between PD29 and PD36 (PD29–31: sham 4.75 ± 1.1 Hz vs. verum 10.51 ± 1.95 Hz, *p* = 0.014; PD34–36: sham 3.51 ± 0.58 Hz vs. verum 7.65 ± 2.1 Hz, *p* = 0.054; see Figures 5B–D). Adding PTX to the bath solution had little effect on sPSC frequency in general, indicating that the synaptic currents measured at −80 mV were mostly mediated by excitatory inputs. Significance levels for differences between verum and sham-treated animals persisted (PD29–31: sham 2.9 ± 0.75 Hz vs. verum 9.25 ± 2.58 Hz, *p* = 0.015) or slightly improved (PD34–36: sham 4.07 ± 0.85 Hz vs. verum 8.67 ± 2.1 Hz, *p* = 0.04).

### iTBS Effects on sEPSC Amplitude

In addition to frequency, we analyzed the amplitude distribution of sEPSCs in FSI of sham and verum iTBS treated animals (PTX added to bath solution). Comparison of sham and verum treated groups using Wilcoxon rank-sum test indicates significantly increased sEPSC amplitudes in all age groups (*p* < 0.001) with a stronger increase evident for the oldest group (PD40–42, see cumulative amplitude plots of Figures 5E–G). Measured at 50% of cumulative amplitudes, sEPSCs amplitudes were on average 0.5 pA, 0.5 pA and 2 pA larger for the verum groups PD29–31, PD34–36 and PD40–42, respectively, compared to corresponding sham groups. At 75% cumulated amplitudes the differences increased to 1.5 pA, 1.5 pA and 2.5 pA, respectively. A separate comparison of the sEPSC amplitudes of the sham-groups also revealed a significant increase with age (*p* = 0.047, Kruskal-Wallis test for comparison of all groups; *p* < 0.001 for pair-wise comparison of age-groups with Wilcoxon rank-sum test, Figure 5H). Measured at 50% of cumulative amplitudes, sEPSCs somewhat decreased between PD29–31 and PD34–36 (−1.5 pA) but increased between PD34–36 and PD40–42 (+2.5 pA). Corresponding differences at 75% cumulated amplitudes are −0.5 pA and +2 pA, respectively.

## Discussion

### Summary of Findings

Our current *ex vivo*—*in vitro* studies demonstrate that iTBS applied via TMS to the rat brain alters the electrical properties of cortical FSI compared to age-matched sham-stimulated controls. We further show that the iTBS effect varies in an age-dependent fashion during early cortical development and that the strongest effects were evident during the phase of cortical critical period when FSIs mature in an activity-dependent fashion (see Hensch, 2005). The major iTBS effects seen in the acute brain slice preparations were: (1) an enhanced excitability of FSIs between PD 29 and 36 (PD29–36), evident as an increased action potential firing frequency in response to small depolarizing current injections, accompanied by a less hyperpolarized membrane potential; and (2) an enhanced activity level of the cortical network, evident by increased frequency of sEPSCs in FSIs. An iTBS-induced increase in sEPSC amplitude was stronger for animals older than 33 days when sEPSC amplitudes generally increased with age. Passive cell membrane properties like membrane input resistance, capacitance, time constant and the action potential threshold did not change after iTBS.

### Possible Cellular Mechanisms

Although we cannot demonstrate discrete cellular mechanisms of these changes in FSI and network excitability, our results demonstrate that the iTBS protocol applied via TMS to the rat neocortex is able to affect the electrical properties of FSIs in addition to the biochemical changes demonstrated previously. Our *in vivo* TMS studies revealed that iTBS applied with a total of 1800 pulses (3 blocks at 600 pulses at 15 min intervals) strongly reduced the cortical expression of PV in adult (Benali et al., 2011; Hoppenrath and Funke, 2013; Volz et al., 2013; Mix et al., 2014) but also in younger, but more than 32-day-old rats (Mix et al., 2015). Usually, PV was strongly reduced in 40–50% of the cells compared to control or sham-stimulated animals. Those cells with reduced PV expression still appeared vital as demonstrated by the normal pattern of the PNNs, the unchanged expression of Kv3.1b-type potassium channels and the integrity of synaptic connections (Benali et al., 2011). Also the electrophysiological data obtained in the current study indicate no pathological state of FSIs in iTBS-treated animals. Almost all Cy3-WFA pre-labeled neurons showed the typical non-adapting fast-spiking behavior to constant depolarizing current injections, with spike train frequencies between 30 and 200 Hz (Cauli et al., 1997; Uematsu et al., 2008; Orduz et al., 2013). Also an adaptation coefficient scattering somewhat around 0.88 is typical for FSI (see Imbrosci et al., 2014). Spike width appeared small (<0.5 ms; at half height) but could not be reliably measured with the sampling frequency related to the current injection protocol (2.73 kHz). However, FSIs recorded from iTBS-treated rats showed on average a higher excitability to weak depolarizing current injections. It is likely that the enhanced excitability is a result of the reduced PV expression. Orduz et al. (2013) recently demonstrated a very similar relationship between PV expression and induced spike sequences in FSIs of young (PD18–24) PV-knockout mice (PV^−/−^). Compared to wild-type controls, PV^−/−^ mice exhibited a 100–200% increase in spike-firing frequency particularly at low current injections (100–200 pA) while no mean difference was found with strong current injection (380 pA). This difference disappeared if EGTA, a calcium chelator exhibiting similar calcium-buffer kinetics as PV, was added to the patch-pipette solution during recordings of FSIs with PV^−/−^ phenotype. The authors of this study further demonstrated that a faster deactivation of SK-type calcium-dependent potassium channels is fundamental to the increased firing frequency of FSIs of the PV^−/−^ phenotype (see also Gall et al., 2003; Bischop et al., 2012) and related to the changed calcium-buffering capacity of the cells missing PV. Although the studies of Orduz et al. (2013) were done on striatal neurons of mice aged PD29 onwards and therefore, cannot be transferred to neocortical FSIs in a one-to-one fashion, their findings are strong indications that the calcium-buffering capacity of PV regulates the excitability and activity rate of FSIs. Unfortunately, we were unable to confirm that the cells recorded in slices from iTBS-treated rats actually had reduced PV expression, because subsequent quantification of cytosolic PV concentration appeared to be unrealistic. Nevertheless, due to our previous immunohistochemical studies on rats of matching age and strain (Mix et al., 2015) the likelihood of recording a neuron with reduced PV expression (but normal PNNs) is 40–50% in iTBS-treated rats compared to almost 0% in sham-treated animals, meaning that the differences between verum and sham-stimulated animals may be even stronger if we would have recorded only neurons certainly missing the PV expression.

### Cortical Network Activity

Within the same range of age, iTBS caused an increase in sEPSCs in FSIs indicating an increased cortical network activity. This finding is in agreement with the idea of increased cortical excitability after iTBS according to human (Huang et al., 2005; Di Lazzaro et al., 2010) but also animal studies (Benali et al., 2011; Thimm and Funke, 2015). Although spontaneous excitatory input at a rate of about 5 Hz appears to be insufficient to result in a steady 10 mV depolarization of the membrane potential of FSIs compared to the 2–3 Hz input in the control condition, we cannot exclude that increased EPSC frequency following iTBS contributes to the cell’s increased excitability. A study of Chance et al. (2002) demonstrated that high-frequency synaptic noise (but balanced for excitation [7000 Hz] and inhibition [3000 Hz]) can modulate the cells response gain to driving inputs. However, the effects on response gain considerably differed from those we observed: instead of increased steepness of the stimulus-response curve we found increased responses to weak stimuli. A more detailed quantification of the contribution of Ca^2+^-buffering by PV and synaptic input to the cell’s excitability could be achieved by blocking the latter. However, our first measurements aimed at showing the iTBS effect on FSIs for a condition close to the physiological state. The effect of increased spontaneous activity after iTBS was weaker but not completely absent in the older animals. Also previous studies applying iTBS to anesthetized adult rats (2–3 month) revealed an increase in cortical excitability expressed as increased spontaneous and sensory stimulation driven neuronal activity (Benali et al., 2011; Thimm and Funke, 2015; see also Supplementary Figure 2). On the other hand, sEPSC amplitudes in the *ex vivo*—*in vitro* recordings of FSIs increased not only after iTBS primarily in the elder animals (PD 40–42) but progressively increased also with age, likely a sign of maturing excitatory synapses on FSIs.

### Age and Developmental Cortical State

The rTMS studies in adult rats indicate a disinhibitory effect of iTBS since sensory responses of the somatosensory cortex increased (Benali et al., 2011; Thimm and Funke, 2015). In addition, rats showed a better tactile learning performance after iTBS which was correlated with decreased PV expression after iTBS and re-instatement of PV expression after learning (Mix et al., 2010). These findings are in line with improved human tactile and motor performance when cortical GABA content is altered (Dinse et al., 2003; Kim et al., 2014; Heba et al., 2016) and fit the hypothesis of disinhibition-guided learning (Letzkus et al., 2015). In our current study, we found no indications of reduced activity in FSIs after iTBS but an increased excitability to weak depolarizing stimuli. It is very likely that the physiological state of the FSIs during the cortical critical period very much differs from the adult state when exhibiting an activity-driven episode of maturation that goes along with synaptic pruning, potentiation of surviving synapses and restriction of neuronal plasticity (Hensch, 2005; Sale et al., 2010) with growth of the PNNs (Köppe et al., 1997) frequently surrounding PV^+^ neurons (Härtig et al., 1992, 1999; Wegner et al., 2003). In particular, inhibition mediated via the PV^+^ FSIs appears to be a key mechanism in this process (Huang et al., 1999; Chattopadhyaya et al., 2004) since it reduces the ratio of uncorrelated spontaneous input to well correlated synaptic activity driven by sensory inputs (Maffei et al., 2004, 2006; Toyoizumi et al., 2013; Chen et al., 2014). PV^+^ interneurons apparently exhibit a higher degree of responsiveness and plasticity to driving inputs at this stage, resulting in a stronger change in excitability between PD 29–38 also when cortical inputs are artificially activated via high-frequency rTMS. At the same age iTBS was found to reduce the expression of PV which did not occur in younger animals (Mix et al., 2015). Since the way we applied rTMS favors the activation of callosal axons rather than short-projecting cells within the gray matter (low intensity mediolateral oriented electric field), FSIs can be excited only trans-synaptically *via* collaterals of the callosal axons (see Benali et al., 2011; Funke and Benali, 2011). As long as these synapses are still immature, no effect can be expected. Later, but still during the early phase of critical period, these synapses will be strengthened in an activity-dependent manner while inhibition is still weak (Kuhlman et al., 2013). A lack of sensory activity as during dark-rearing of the animals impairs this maturation process (Fagiolini et al., 1994; Pizzorusso et al., 2000). We could recently show that iTBS applied during this critical period in dark-reared rats is able to substitute for the missing visual input, restoring the animal’s visual performance (Castillo-Padilla and Funke, 2016). This was accompanied by increased cortical levels of BDNF, a possible mediator of improved functional maturation of FSI (Gianfranceschi et al., 2003; Jiao et al., 2011).

### Translational Aspects

Non-invasive brain stimulation appears to be a promising tool for treating neurological and psychiatric disorders (Lefaucheur et al., 2014). Of particular interest in this respect is the chance to modulate neuronal plasticity processes, e.g., by re-instating a juvenile-like state of cortical plasticity as during cortical critical period. Attempts have been made to modify cortical inhibition by varying the sensory environment (Baroncelli et al., 2010a,b, 2011), by pharmacological means (Gianfranceschi et al., 2003; Maya Vetencourt et al., 2008; Baroncelli et al., 2010b; Harauzov et al., 2010) and even by transplanting precursor cells of inhibitory interneurons to the adult cortex (Southwell et al., 2010; Davis et al., 2015) to achieve such a state. Also cortical injury appears to trigger such processes (Imbrosci et al., 2014; Nahmani and Turrigiano, 2014). The findings of our current and recent studies (Castillo-Padilla and Funke, 2016; Mix et al., 2015) with iTBS applied during cortical critical period indicate that non-invasive brain stimulation could affect early cortical developmental plasticity by modulating the activity of FSIs. This would be of potential benefit if activity-dependent maturation of FSIs is impaired due to sensory deprivation or disturbed glutamatergic input as discussed for developmental causes of neuropsychiatric diseases (Taylor and Tso, 2015). However, adequate work in children is missing and those in animal models are rare so far, demanding further studies on this important aspect. A couple of studies—including the use of TMS—indicate that cortical excitability and plasticity change with age accompanied by changes in the impact of inhibitory systems (Rogasch et al., 2009; Todd et al., 2010; Bashir et al., 2014). A recent study using iTBS, however, showed no clear age-dependent variations but a high variability of TMS effects in general (Dickins et al., 2015). Besides general processes of cellular aging, experience-dependent changes in network wiring strategies are discussed as contributing factors (e.g., Bernard and Seidler, 2012). Therefore, cortical networks of children are apparently differently responsible to natural and artificial cortical stimulation and also the effects of rTMS may differ. So far, little is known about iTBS effects in children but it appears to be safe since adverse effects are rare and do not outnumber those of seen in adults (Hong et al., 2015; Pedapati et al., 2015).

A further challenge is the analysis of the state-dependence of brain stimulation and the comparison of different stimulation protocols. The two established rTMS theta-burst protocols iTBS and cTBS have initially been shown to induce opposite changes in cortical excitability (Huang et al., 2005; Di Lazzaro et al., 2010), recent studies however demonstrate a strong inter-individual variation which obviously relates to varying states of cortical network connectivity (Hamada et al., 2013; Nettekoven et al., 2015) and homeostatic plasticity (Karabanov et al., 2015). Although we found partly opposite effects of iTBS and cTBS in our rat studies on neuronal activity marker expression (Trippe et al., 2009; Benali et al., 2011) and learning performance (Mix et al., 2010), we also found a clear strain-dependent variation especially of the cTBS effect (Mix et al., 2014). The effects of cTBS appear to change also if applied with different numbers of pulses (Gentner et al., 2008; Gamboa et al., 2010) and if applied repeatedly (Gamboa et al., 2011; Thimm and Funke, 2015). Therefore, we focused our studies on the less variable iTBS effect. Nevertheless, further studies on the age-dependence of cTBS effect are required to better understand the reasons for the variable stimulation effects.

## Author Contributions

KH conducted the experiments, analyzed the data and wrote a first version of the manuscript as part of her PhD thesis. The experiments were carried out in the Department of Neurophysiology, Ruhr-University Bochum. KF and WH designed the study and revised the manuscript. KF proofed the data analysis and statistics, and helped with the design of the figures. WH prepared the Cy3-WFA for staining of perineuronal nets in the living slice. All authors approved the final submission and agree to be accountable for all aspects of the work in ensuring that questions related to the accuracy or integrity of any part of the work are appropriately investigated and resolved.

## Funding

This study has been supported by grants from the Deutsche Forschungsgemeinschaft (DFG) to KF (SFB 874, TP A4 and FU256/3-2) and is also a matter of the competence network on neuropsychiatric diseases of the German Federal Ministry of Education and Research (BMBF, with funding to KF for project: 01EE1403B).

## Conflict of Interest Statement

The authors declare that the research was conducted in the absence of any commercial or financial relationships that could be construed as a potential conflict of interest.

## Abbreviations

ACSF: artificial cerebrospinal fluid
BDNF: brain-derived neurotrophic factor
Cy3-WFA: *Wisteria floribunda* agglutinin conjugated to carbocyanine 3
EGTA: ethylene glycol tetraacetic acid
FSIs: fast-spiking interneurons
GAD67: 67kD isoform of glutamic acid decarboxylase
iTBS: intermittent theta-burst stimulation
LTD, LTP: long-term depression/potentiation
PBS: phosphate buffered saline
PD: postnatal day
PNNs: perineuronal nets
PV: parvalbumin
PTX: picrotoxin
QX-314: (N-Ethyllidocaine chloride)
rTMS: repetitive transcranial magnetic stimulation
TMS: transcranial magnetic stimulation.
