# Supplementary material for: Intermittent Theta-Burst Transcranial Magnetic Stimulation Alters Electrical Properties of Fast-Spiking Neocortical Interneurons in an Age-Dependent Fashion
Source: Front Neural Circuits. 2016 Mar 30;10:22. doi: 10.3389/fncir.2016.00022 (PMC4811908; doi:10.3389/fncir.2016.00022)
Supplement: Supplementary file 1 [file DataSheet_1.pdf]

Supplementary material

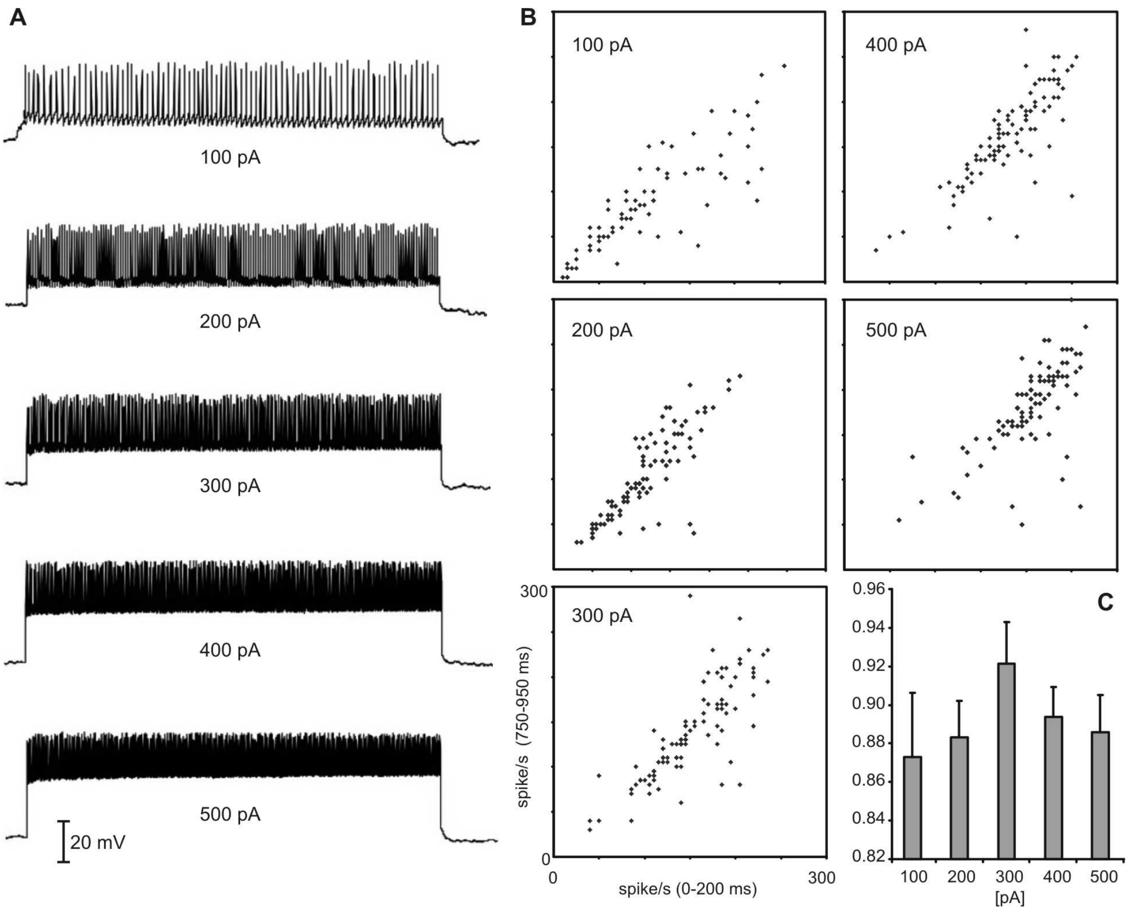

Supplementary Figure 1

Supplementary Figure 1: Firing characteristics of Cy3-WFA pre-labelled neurons. (**A**) Voltage traces of representative non-adapting spike sequences evoked by current injections of 1s duration ranging between 100 pA and 500 pA. (**B**) Quantification of spike-train frequency and adaptation behaviour. Dot raster diagrams plot firing frequency between 750 and 950 ms following onset of current injection versus initial firing frequency (0-200 ms) for each of the five different current injections (100-500 pA). (**C**) The bar diagram to the lower right shows the corresponding mean adaptation coefficients (spikes/s 750-950ms / spikes/s 0-200ms; +/- SD) of all 114 neurons included in this analysis.

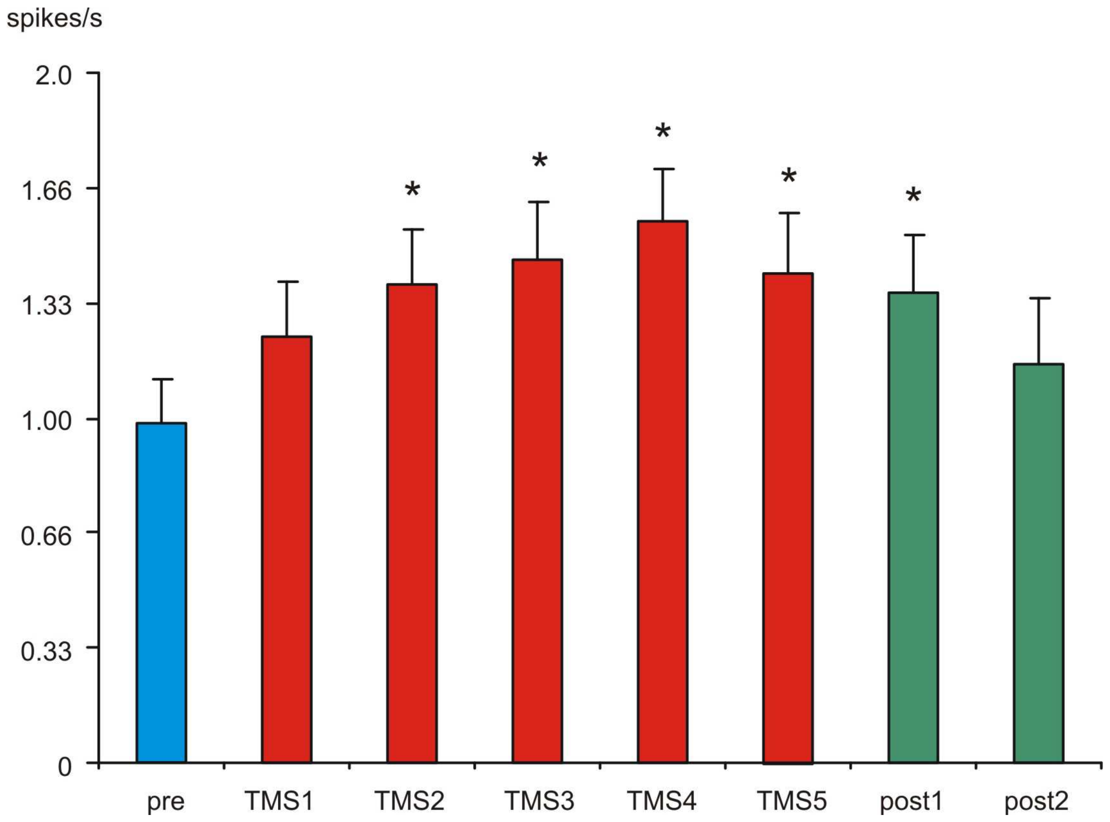

Supplementary Figure 2

Supplementary figure 2 shows rates of spontaneous multi-unit spiking activity recorded *in vivo* from barrel cortex of urethane anesthetized adolescent rats (10-12 weeks old). Spike rates were obtained before (blue), after each of 5 TMS applications (one block of iTBS = 600 pulses) (red) as well as between 60 and 120 min (post1) and between 120 and 180 min (post2) after the last TMS block (green). TMS blocks were applied at 20 min intervals and spontaneous activity between TMS blocks was analyzed from 7 min post TMS on up to the next TMS block. Data were obtained from recordings fundamental to the publication Thimm & Funke (2015) but now analyzing spontaneous multi-unit activity between episodes of whisker stimulation. * $p < 0.05$ compared to "pre" condition (Dunnett's test).
